# Supplementary material for: TRAP1 Is Expressed in Human Retinal Pigment Epithelial Cells and Is Required to Maintain their Energetic Status
Source: Antioxidants (Basel). 2023 Feb 4;12(2):381. doi: 10.3390/antiox12020381 (PMC9952053; doi:10.3390/antiox12020381)
Supplement: Supplementary file 1 [file antioxidants-12-00381-s001.zip › antioxidants-2144681-supplementary.pdf]

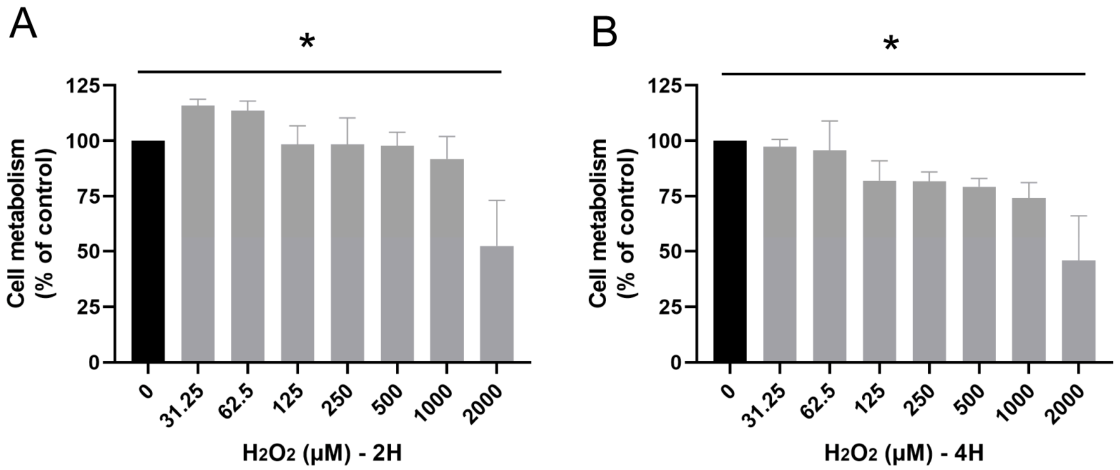

Figure S1. Assessment of cell metabolism of ARPE-19 cells upon challenge with hydrogen peroxide. ARPE-19 cells were treated with different doses of H_2O_2 (0; 31.25; 62.5; 125; 125; 250; 500; 1000 and 2000 μM) for 2 h (**A**) and 4 h (**B**). NS: Non-stimulated (black bars); ST: stimulated with H_2O_2 (grey bars). The values are presented as a percentage of the control (non-treated cells). Cell metabolism was assessed using the resazurin assay. Four independent experiments were performed (n = 4). Statistical significance was calculated by using the Mann–Whitney U test. Statistically significant values: * $p < 0.05$. In the graphs, all values are expressed as mean ± standard error of the mean (SEM).
